# Seronegative Immune-Mediated Necrotizing Myopathy: A Case Report

**DOI:** 10.7759/cureus.27824

**Published:** 2022-08-09

**Authors:** Shriya Patel, Mohammad Abu-Abaa, Feryal Mousavi

**Affiliations:** 1 Department of Internal Medicine, Rowan University School of Osteopathic Medicine, Stratford, USA; 2 Department of Internal Medicine, Capital Health Regional Medical Center, Trenton, USA

**Keywords:** malignancy risk, inflammatory myositis, statin use, corticosteroid treatment, immune-mediated necrotizing myopathy

## Abstract

Idiopathic inflammatory myopathies (IIMs) are a group of chronic autoimmune disorders characterized by proximal skeletal muscle weakness. One subtype of the IIMs is immune-mediated necrotizing myopathy (IMNM). IMNM can be further classified according to its autoantibody presence, including anti-3-hydroxy-3-methylglutaryl-coenzyme A reductase (HMGCR), anti-signal recognition particle (SRP), and seronegative.

Here, we describe the case of a 61-year-old Caucasian female with a prior history of distant lung cancer and current statin use presenting with a subacute onset of bilateral proximal lower extremity muscle weakness and markedly elevated creatinine kinase (CK) and amino transaminases. In the acute inpatient setting, she underwent successful treatment with corticosteroids that were eventually discontinued and replaced with azathioprine three months after hospital admission. At that point, she had attained a 60% increase in muscle strength.

## Introduction

Immune-mediated necrotizing myopathy (IMNM) is an autoimmune-mediated muscle disease that leads to severe proximal muscle weakness, markedly elevated creatinine kinase (CK), and myofiber necrosis with minimal to no inflammatory cell infiltrates on muscle biopsy [1]. The prognosis of IMNM is generally worse than other types of myositis [1]. IMNM is further classified into three subtypes based on autoantibody serology: anti-3-hydroxy-3-methylglutaryl-coenzyme A reductase (HMGCR), anti-signal recognition particle (SRP), and seronegative [1]. These conditions, which have mild variations in clinical severity and histopathological features, have an incidence rate of 3-7 cases per 100,000 persons a year [2]. Of the three subtypes, seronegative IMNM is the most poorly described and least common [1]. As a result of the rarity of IMNM, most cases tend to be misdiagnosed as polymyositis (PM). Also, there are no randomized clinical trials to help guide therapy for patients with seronegative or seropositive IMNM. Thus, therapy is mostly based on clinical case series. Here, we aim to shed light on successful treatment strategies for seronegative IMNM with steroid monotherapy and an eventual transition to a steroid-sparing agent. We also aim to review the current literature on IMNM.

## Case presentation

A 61-year-old Caucasian female presented to the ED with five months of progressively worsening bilateral proximal lower extremity weakness, along with acute onset abdominal pain and non-bloody diarrhea. In the week prior to presentation, her lower extremity weakness rapidly progressed to a point where she was not able to ambulate and could not raise herself from a toilet. This was associated with a significantly decreased oral intake and myalgias. Her past medical history is significant for peripheral artery disease status/post (s/p) femoral-popliteal bypass and stent, obesity, coronary artery disease, hypertension, hyperlipidemia, small fiber peripheral neuropathy, ulcerative colitis without immunosuppression, and a distant history of lung cancer several years ago s/p partial lobe resection. Of note, home medications include rosuvastatin 40 mg. She denied inciting trauma, strenuous exercise, shortness of breath, chest pain, or fever. Physical exam revealed hyperalgesia and altered sensation to light touch in bilateral L3-L4 dermatomes. Muscle strength testing graded by the American Spinal Injury Association (ASIA) impairment scale demonstrated weakness in hip flexion (3/5 on the left, 4/5 on the right) but was otherwise within normal limits. Upper extremity muscle strength and sensation were grossly intact. Deep tendon reflexes (DTRs) of bilateral upper and lower extremities were +2/4. There was no evidence of ascites, abdominal striae, or jaundice. A thyroid examination was unremarkable. There were no signs of a heliotrope rash on the face, Gottron papules on the hands, or erythema. There was no muscle wasting in the upper or lower extremities. Gait evaluation was deferred due to the patient's safety. 

**Figure 1:**
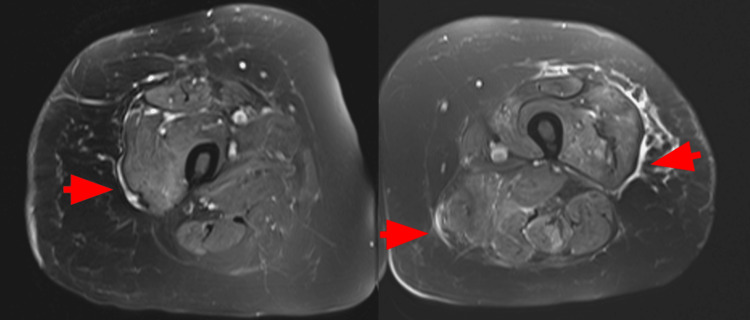
An axial T2-weighted fat sequence MRI image of bilateral femurs shows diffuse muscle edema (red arrow).

The patient was admitted for further workup and evaluation. Initial laboratory evaluation is summarized in Table 1. Urinalysis was positive for large amounts of blood and 10-25 RBCs/high-powered field. CT of the abdomen and pelvis showed findings that suggested colitis and a significant stool burden, and there was no evidence of hepatobiliary pathology. MRI of the lumbar spine demonstrated degenerative changes but did not show any evidence of spinal cord compression, neoplasm, or cauda equina syndrome. Electromyography (EMG) was not performed per the patient's request. T2-weighted fat sequence MRI of bilateral femurs demonstrated mild muscle edema (Figure 1). A muscle biopsy from the right calf was performed. The findings showed active myopathy characterized by scattered necrotic and regenerative myofibers and chronic histio-lymphocytic inflammatory infiltrates closely associated with necrotic foci. There were no significant histopathological changes on skin biopsy.

**Table 1:** Initial serum laboratory evaluation.

Electrolyte	Current result	Reference range
Sodium (Na)	140 mmol/L	137-145
Potassium (K)	3.3 mmol/L	3.5-5.1
CK	9641 unit/L	24-204
Aspartate aminotransferase (AST)	404 unit/L	14-36
Alanine aminotransferase (ALT)	258 unit/L	0-34
Alkaline phosphatase	95 unit/L	38-126
Blood urea nitrogen (BUN)	45 mg/dL	9-20
Creatinine	1.65 mg/dL	0.66-1.25

Radiology findings

Because this initial clinical presentation was presumably consistent with statin-induced myopathy, we immediately discontinued rosuvastatin and performed a full myopathic workup. Unfortunately, subsequent myositis serology testing demonstrated negative findings, including anti-HMGCR and anti-SRP autoantibodies. However, she did have a mild elevation in anti-proteinase 3 antibody and alpha-1 antitrypsin. Notably, primary sclerosing cholangitis (PSC) and autoimmune hepatitis (AIH) autoantibodies were negative on serology. The primary team immediately started her on IV fluids and methylprednisolone 40 mg twice a day. Response to treatment was assessed with down-trending CK levels over two weeks. She was then transitioned to prednisone 40 mg daily and discharged to a subacute rehabilitation facility. At a two-week follow-up visit in the outpatient rheumatology clinic, she endorsed over a 50% improvement in muscle strength while on prednisone 40 mg daily. She was eventually tapered to 15 mg and then to 10 mg while endorsing over 60% improvement in muscle strength and continuing at-home physical therapy. Three months after hospital discharge, she self-tapered off of prednisone and was started on azathioprine 100 mg daily.

## Discussion

IMNM is a rare subtype of autoimmune IIM. It is characterized by acute or subacute proximal muscle weakness, rare extra-muscular involvement, and myofiber necrosis with minimal inflammatory infiltrate on muscle biopsy [1,3-5]. Although the exact incidence is under debate, the literature suggests that IMNM affects about 3-7 in 100,000 people yearly in the United States, with a female predominance [2]. IMNM has been associated with viral infections, connective tissue diseases, malignancy, and certain toxins [6]. IMNM can be further characterized by its associated autoantibodies, including anti-HMGCR, anti-SRP, and seronegative [1]. Each subtype of IMNM has mild variations in clinical features, severity, and histopathological findings. Anti-SRP patients present with very high CK levels, and only a minority have extramuscular manifestations (rash, lung involvement) [4]. Younger patients were found to have a worse outcome in both anti-SRP and anti-HMGCR IMNM [4-5]. Compared to patients with anti-HMGCR IMNM, those with anti-SRP IMNM have a more severe and active disease even after treatment [4]. Anti-HMGCR patients also have rare dermatologic or pulmonary involvement [5].

The hallmark clinical feature of IMNM is proximal muscle weakness, and the presence of skin or lung involvement should prompt consideration of other subtypes of inflammatory myopathy [1,3-5]. Compared to anti-HMGCR and anti-SRP myopathy, seronegative IMNM has the highest association with malignancy [1,7-8]. It has been found as a paraneoplastic process in the setting of GI, lung, and breast malignancies [7], occurring within three years before or after the diagnosis. There are few cases of paraneoplastic necrotizing myopathy in the literature, with the earliest reports described in 1998 [7]. Importantly, this association with malignancy is likely not as strong as in those with anti-TIF1g-positive dermatomyositis [1,8-10]. In addition, one recent study of 152 patients showed no increased risk of malignancy among those with IMNM [11]. No evidence-based guidelines indicate what cancer screening should be performed in newly diagnosed myositis patients, including those with IMNM [1]. Nonetheless, it is recommended to obtain chest and abdominal CT scans and age- and gender-appropriate cancer screening [1]. In the case described, our patient's malignancy screen was unyielding. However, her distant history of cured lung cancer raises the suspicion of a long latency period associated with IMNM. 

Autoantibody testing is a critical method of classifying IMNM patients into its three subtypes. Anti-SRP autoantibodies are screened by techniques such as enzyme-linked immunosorbent assay (ELISA), line blot assay, and anti-HMGCR antibodies usually screened by ELISA [1]. Anti-HMGCR autoantibodies should only be screened for in patients with a necrotizing muscle biopsy or with a high pretest probability of anti-HMGCR myopathy, including those with significant CK elevations and lack of improvement in muscle weakness after statin discontinuation [1]. Our patient met both of these criteria for anti-HMGCR autoantibody screening, and her results were unremarkable, prompting concern for seronegative IMNM. 

In contrast to patients with other subtypes of myositis, those with IMNM have the highest CK elevations, which is correlated to disease activity [1]. Interestingly, muscle enzyme elevations may precede the development of muscle weakness in IMNM, and serum CK levels often decline weeks to months before muscle regeneration and strength recovery after starting treatment [1]. For this reason, the authors of one report suggested not to escalate therapy once the serum CK has normalized [1]. Muscle MRI assists with managing IMNN but has limited value in diagnosis because it poorly differentiates IMNM from other types of myositis [12]. Regardless, MRI findings in active IMNM include generalized muscle edema, muscle atrophy, and irreversible fatty replacement of muscle [1]. While the specific characteristics in seronegative IMNM have not yet been described, the findings in anti-SRP myopathy involve higher rates of atrophy and fatty replacement, consistent with a more severe myopathy [1]. Muscle biopsy findings in IMNM show prominent necrosis and regeneration of muscle fibers, along with a key feature of mild or absent inflammatory infiltrates. Histopathological differences between anti-SRP and anti-HMGCR include more severe necrosis and regeneration in anti-SRP, sarcolemmal expression of MHC-1 in isolated fibers in anti-SRP, and in clustered fibers in anti-HMGCR, and three times greater expression of sarcolemmal C5b-9 in anti-HMGCR compared to anti-SRP [13]. The detailed pathologic features of seronegative IMNM have not yet been described in the literature [1,13].

There are currently no documented randomized clinical drug trials pointing to an effective treatment for IMNM. However, systemic glucocorticoids are the mainstay of initial treatment for IIMs in general [14-15]. Glucocorticoid-sparing agents, like methotrexate and azathioprine, can be administered concomitantly in patients with moderate-to-severe IIMs [14]. Treatment options for those with the severe disease include rituximab, IV immunoglobulin (IVIG), mycophenolate mofetil, and cyclophosphamide [14-15]. Patients with seropositive (anti-SRP or anti-HMGCR) IMNM have been shown to have mixed results with steroid monotherapy [16]. In this case, we immediately initiated high steroid treatment prior to IMNM biopsy confirmation. This early and aggressive strategy proved to be effective at our patient's three-month follow-up examination, where she showed at least a 60% regain in muscle power. One case report demonstrated a successful reduction in CK with the administration of high-dose glucocorticoids and a pulse dose of IVIG in a patient with seronegative IMNM [17]. This case supports the use of early and aggressive administration of high-dose steroids in patients with seronegative IMNM, with an eventual taper of glucocorticoids and transition to a steroid-sparing agent. The importance of starting intense immunosuppressant therapy early is to offset the possibility of fatty replacement of muscle tissue [1]. Besides medical therapy, all patients with myositis must receive long-term physiotherapy and rehabilitation [18]. Thus, a multidisciplinary approach to management and treatment is necessary. This case is unique because our patient had a history of current statin exposure, along with findings of IMNM on muscle biopsy, initially prompting a diagnosis of statin-induced IMNM. While our patient had a negative anti-HMGCR antibody titer, her prior history of statin use may suggest an overlap in the pathogenesis of anti-HMGCR and seronegative IMNM [17]. This illustrates that not every patient with a history of prior or current statin exposure who presents with signs and symptoms of active myopathy will have a positive HMGCR autoantibody. In addition, her distant history of malignancy may have been another contributing factor towards her diagnosis of seronegative IMNM. Unfortunately, there is a significant percentage of IMNM patients who have persistent muscle weakness despite intense immunosuppressant management [1,3-5]. Even with treatment, up to half of seropositive IMNM patients experience continued muscle weakness after two years of treatment, and a younger age of onset is associated with worse prognosis [1].

## Conclusions

IMNM is a type of IIM characterized by severe proximal muscle weakness. Seronegative IMNM is one subtype that is not associated with either anti-SRP or anti-HMGCR autoantibodies and has a more significant association with malignancy. It is characterized by prominent myofiber necrosis on muscle biopsy, and minimal-to-absent lymphocytic inflammatory infiltrates. Early and prompt recognition is critical to prevent long-term and irreversible damage; however, most patients do not regain full muscle strength even after treatment. While the literature supports an initial regimen of a steroid and steroid-sparing agent for the treatment of IMNM, this case report demonstrates that steroid monotherapy with eventual transition to a steroid-sparing agent, along with physical rehabilitation, maybe a beneficial approach for the long-term management of these patients.
